# Supplementary material for: Crystal structure of PfRh5, an essential P. falciparum ligand for invasion of human erythrocytes
Source: eLife. 2014 Oct 8;3:e04187. doi: 10.7554/eLife.04187 (PMC4356141; doi:10.7554/eLife.04187)
Supplement: Figure 2—source data 1. — DOI: http://dx.doi.org/10.7554/eLife.04187.007 [file elife04187s001.docx]

**Figure 2 – source data.**

**Data collection and refinement statistics of Rh5 and Rh5_KI.**

|  | Native | KI Derivative |
| --- | --- | --- |
| **Data collection** |  |  |
| Space group | P2_1_2_1_2_1_ | P2_1_2_1_2_1_ |
| Cell dimensions |  |  |
| *a*, *b*, *c* (Å) | 53.98, 86.26, 114.83 |  |
| Resolution (Å) | 50-2.18 (2.21-2.18) | 50-3.46(3.74-3.46) |
| *R*_merge_ (%) | 35.2 (530.8) | 32 (100.9) |
| *<I* / σ*I>* | 5.44 (0.52**)** | 7.87 (3.47) |
| Completeness (%) | 99.9 (99.9) | 100 (100) |
| Redundancy | 7.2 (7.3) | 4.8 (4.3) |
| *CC*_1/2_^*^ | 99.3 (16.4) | 97.6 (76.5) |
| σ**_Ano_**  Centric phasing power^#^  Acentric phasing power  Number of sites  **Refinement** | **-** | 0.956 (1.020)  0.57  0.73  3 |
| Resolution (Å) | 45.76-2.18 (2.26-2.18) |  |
| No. reflections | 28698/1454 |  |
| *R*_work_ / *R*_free_ | 20.0/24.5 (27.1/29.0) |  |
| No. atoms |  |  |
| Protein  Carbohydrate | 2822  14 |  |
| Ligand/ion | 5 |  |
| Water | 82 |  |
| *B*-factors (Å)^2^ |  |  |
| Protein  Carbohydrate | 58.9  67.9 |  |
| Ligand/ion | 67.9 |  |
| Water | 43.7 |  |
| R.m.s. deviations |  |  |
| Bond lengths (Å) | 0.009 |  |
| Bond angles ()  Ramachandran plot ( % )  Most favoured  Allowed  Outlier | 1.06  97.0  2.7  0.3 |  |

^*^ *CC*^(1/2)^ = Pearson correlation coefficient between independently merged halves of the data set ([1](#_ENREF_1)). Highest resolution shell *CC*^(1/2)^ values are significant at the *p* = 0.001 level.

^#^SIR resolution range (Å): 3.61 – 11.16

1. Karplus PA & Diederichs K (2012) Linking crystallographic model and data quality. *Science* 336(6084):1030-1033.
